# Supplementary material for: Increased Bone Marrow Uptake and Accumulation of Very-Late Antigen-4 Targeted Lipid Nanoparticles
Source: Pharmaceutics. 2023 May 27;15(6):1603. doi: 10.3390/pharmaceutics15061603 (PMC10304323; doi:10.3390/pharmaceutics15061603)
Supplement: Supplementary file 1 [file pharmaceutics-15-01603-s001.zip › pharmaceutics-2394248-supplementary.pdf]

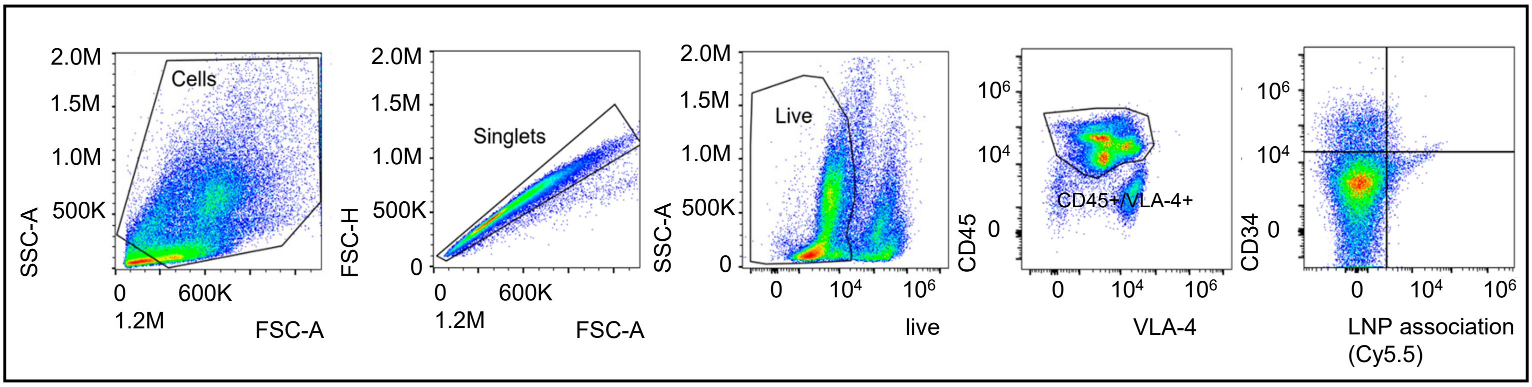

Figure S1. Gating strategy for single bone marrow cells. Bone marrow gating strategy shown for PBS-treated mouse. From left to right: the gating of cells (FSC-A versus SSC-A), singlets (FSC-H versus FSC-A), live cells (SSC-A versus live/dead stain), CD45+/VLA-4+ cells (CD45-A versus VLA-4 marker) and CD34-A versus LNP Cy5.5. The gates were set based on unstained controls.

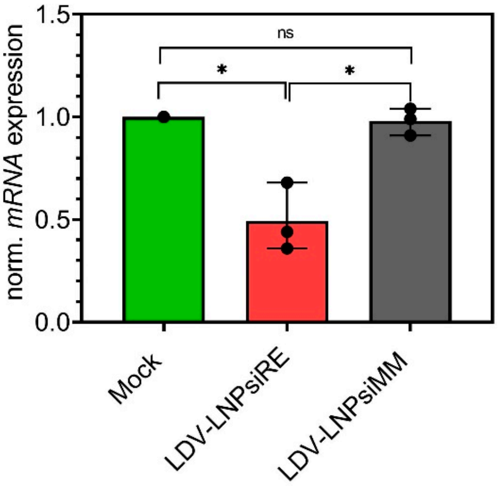

Figure S2. Reduction of *RUNX1/ETO* transcript in PDX. RUNX1/ETO-expressing AML cells were incubated for 24 hours with 4 µg/ml siRNA LNPs followed by qPCR analysis at day 3. This figure displays a subset of the samples from Fig. 1f, samples are here normalized against the ddCt of the mock cells. Mean + range are displayed. Significance was tested by paired Student's t-test *P <0.05, **P <0.001, ***P<0.001, n = 3

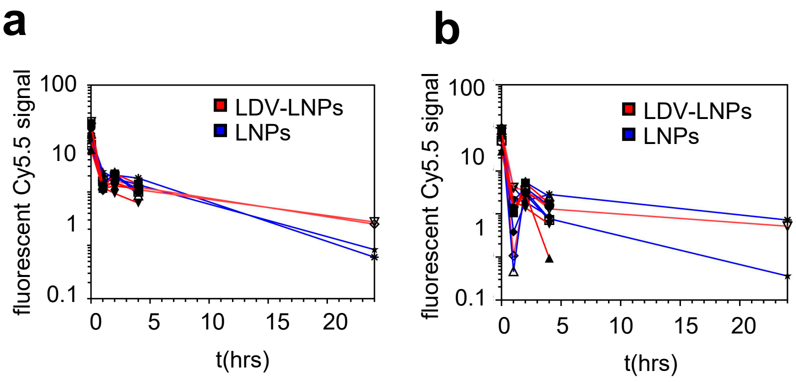

Figure S3. Biodistribution of LDV-LNPs and LNPs in BALB/cAnNCrl mice. (a, b) Circulation time of the LNPs on a log10 scale. Plasma concentration is expressed as the LNP Cy5.5 (a) or siRNA Cy7 (b) fluorescent signal in plasma. At various time points per individual mouse.

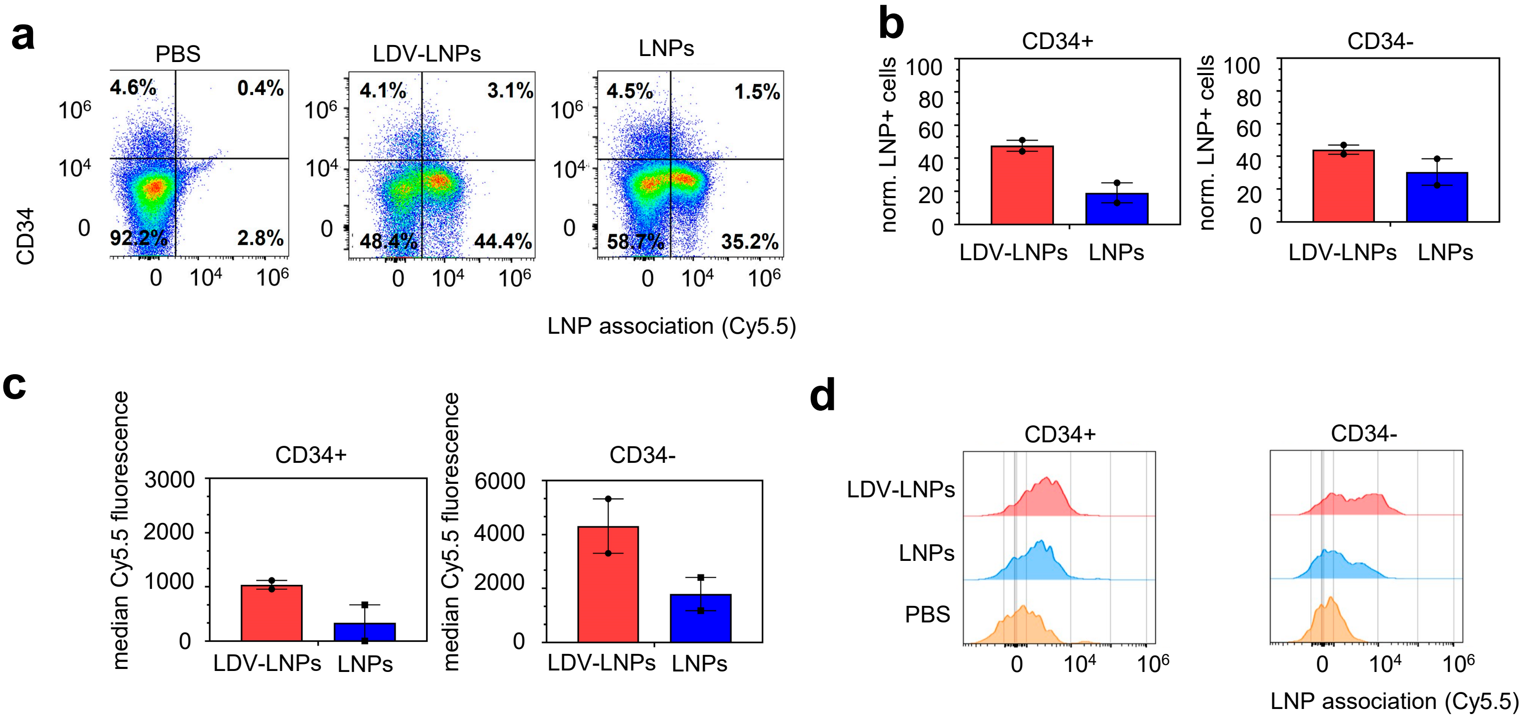

Figure S4. Improved accumulation of LDV-LNPs in HPSC in the bone marrow. (a) Scatter plots showing the CD34 expression and LNP association with CD45+/VLA-4+ cells in the bone marrow after 24 hours for mice treated with PBS, LDV-LNPs or LNPs as determined by multiparameter flow analysis. (b) Bar graphs showing the percentage of LNP+ cells in CD45+/VLA-4/CD34+ (left) or CD45+/VLA-4+/CD34- (right) bone marrow cells as determined by multiparameter flow analysis after 24 hours. (c, d) LNP Cy5.5 association in CD34+ (left) and CD34- (right) bone marrow cells after 24 hours displayed in bar graphs (c) or histograms (d) where LDV-LNPs is red, LNPs blue and control PBS orange. (b, c) Means + ranges are displayed. n = 2.

Table S1. –siRNA sequences and their chemical modifications. F, 2’-fluoronucleoside; OMe, 2’-methoxynucleoside; d, 2’deoxynucleoside; PS, phosphorothioate-. For *in vivo* visualization the sense strand of the siRNA was modified with Cy7 linked via an NHS ester bond. The two swapped nucleotides in the mismatch siRNA control are displayed **bold**.

Name	5’ -> 3’	Supplier
siRE-mod	5’-C$_F$C$_F$U$_F$C$_F$GAAAU$_{OMe}$C$_{OMe}$GU$_{OMe}$AC$_{OMe}$U$_{OMe}$GdAdGdAdT$_{PS}$dT-3’<br>3’-dT$_{PS}$dTGGAGCUUUAGCAUGACUCU-5’	Axolabs
siRE-mod-Cy7	5’-(Lumi-SulfoCy7-NHS)(NHC6)C$_F$C$_F$U$_F$C$_F$GAAAU$_{OMe}$C$_{OMe}$GU$_{OMe}$AC$_{OMe}$U$_{OMe}$GdAdGdAdT$_{PS}$dT-3’<br>3’-dT$_{PS}$dTGGAGCUUUAGCAUGACUCU-5’	Axolabs
siMM-mod	5’-C$_F$C$_F$U$_F$C$_F$GA**AU$_{OMe}$**U$_{OMe}$CG**U$_{OMe}$U$_{OMe}$**C$_{OMe}$UGdAdGdAdT$_{PS}$dT-3’<br>3’-dT$_{PS}$dTGGAGCU**UA**AGC**AA**GACUCU-5’	Axolabs

Table S2. –qPCR primer sequences.

Gene		Sequence (5'-> 3')
GAPDH	Forward	GAAGGTGAAGGTCGGAGTC
	Reverse	GAAGATGGTGATGGGATTTC
RUNX1/ETO	Forward	AATCACAGTGGATGGGCCC
	Reverse	TGCGTCTTCACATCCACAGG
TBP	Forward	CCTAAAGACCATTGCACTTCGT
	Reverse	GTTCGTGGCTCTCTTATCCTCA
